# Lung function decline in relation to diagnostic criteria for airflow obstruction in respiratory symptomatic subjects

**DOI:** 10.1186/1471-2466-12-12

**Published:** 2012-03-22

**Authors:** Reinier P Akkermans, Marvin A Berrevoets, Ivo J Smeele, Annelies E Lucas, Bart P Thoonen, Joke G Grootens-Stekelenburg, Yvonne F Heijdra, Chris van Weel, Tjard R Schermer

**Affiliations:** 1 Department of Primary and Community Care, Radboud University Nijmegen Medical Centre, P.O. Box 9101, 6500 HB Nijmegen, the Netherlands; 2 COPD and Asthma General Practitioner Advisory Group (CAHAG), P.O. Box 3231, 3502 GE Utrecht, the Netherlands; 3 Diagnostiek voor U (DCE), P.O. Box 6274, 5600 HG Eindhoven, the Netherlands; 4 General Practice Laboratory Foundation East (SHO), Velp, the Netherlands; Department of Primary and Community Care, Radboud University Nijmegen Medical Centre, P.O. Box 9101, 6500 HB Nijmegen, the Netherlands; 5 Department of Chest Diseases, Radboud University Nijmegen Medical Centre, P.O. Box 9101, 6500 HB Nijmegen, the Netherlands

**Keywords:** Airflow obstruction, Chronic obstructive pulmonary disease, Diagnosis, Lung function decline, Primary care, Spirometry

## Abstract

**Background:**

Current COPD guidelines advocate a fixed < 0.70 FEV1/FVC cutpoint to define airflow obstruction. We compared rate of lung function decline in respiratory symptomatic 40+ subjects who were 'obstructive' or 'non-obstructive' according to the fixed and/or age and gender specific lower limit of normal (LLN) FEV1/FVC cutpoints.

**Methods:**

We studied 3,324 respiratory symptomatic subjects referred to primary care diagnostic centres for spirometry. The cohort was subdivided into four categories based on presence or absence of obstruction according to the fixed and LLN FEV1/FVC cutpoints. Postbronchodilator FEV1 decline served as primary outcome to compare subjects between the respective categories.

**Results:**

918 subjects were obstructive according to the fixed FEV1/FVC cutpoint; 389 (42%) of them were non-obstructive according to the LLN cutpoint. In smokers, postbronchodilator FEV1 decline was 21 (SE 3) ml/year in those non-obstructive according to both cutpoints, 21 (7) ml/year in those obstructive according to the fixed but not according to the LLN cutpoint, and 50 (5) ml/year in those obstructive according to both cutpoints (p = 0.004).

**Conclusion:**

This study showed that respiratory symptomatic 40+ smokers and non-smokers who show FEV1/FVC values below the fixed 0.70 cutpoint but above their age/gender specific LLN value did not show accelerated FEV1 decline, in contrast with those showing FEV1/FVC values below their LLN cutpoint.

## Background

Chronic obstructive pulmonary disease (COPD) is a disease that is characterized by irreversible and progressive airflow obstruction, and is associated with high morbidity and mortality [1]. COPD is predominantly diagnosed in adults aged well over 40 years. In developed countries cigarette smoking is the main risk factor [2], and accelerated lung function decline is the predominant clinical and prognostic hallmark of the disease [3]. Spirometry is recommended to assess airflow obstruction, i.e. to establish the ratio of the forced expiratory volume in one second (FEV1) and forced vital capacity (FVC). Next, severity of obstruction is quantified by calculation of FEV1 as percentage of predicted value.

For subjects suspected of having the disease current clinical COPD guidelines recommend a fixed FEV1/FVC cutpoint < 0.70 (after administration of a bronchodilator) to decide whether or not airflow obstruction is present [1,4]. However, because lung function physiologically declines with age [5], it has recently been advocated that a correct definition of airflow obstruction should not be based on a fixed cutpoint for all ages, but should take the physiological decline into account [6]. One suggested approach for this is to use lower limit of normal (LLN) cutpoints based on the distribution of FEV1/FVC values in an appropriate reference population, which takes gender and age differences between individuals into account [7,8]. Several recent studies have shown high rates of false-positive interpretations (especially among elderly subjects) when the 0.70 fixed cutpoint is off-set against an age-specific LLN cutpoint [9-14]. Because the majority of COPD patients are diagnosed and managed in primary care [15] and primary care doctors need to differentiate between various underlying causes for the respiratory symptoms a patient presents with (i.e., asthma, COPD, congestive heart failure, and a wide range of other causes), it is especially important for them to know which cutpoint is preferred when assessing the presence (or absence) of airflow obstruction. This is even more important because in elderly patients co-morbid conditions are often present, and misattribution of a patient's symptoms (e.g., dyspnoea) to COPD could lead to inappropriate or delayed treatment.

We previously reported on the use of different criteria to diagnose airflow obstruction in subjects who present with respiratory symptoms in primary care [16]. To date, little longitudinal research has been published to establish the course of clinical markers of COPD prognosis in relation to the recommended diagnostic criteria for airflow obstruction [17-19]. An influential review acknowledged that overestimation of airflow obstruction with the fixed FEV1/FVC ratio becomes more problematic with increasing age, but also stated that the incremental benefits of changing the recommendation to use the fixed 0.70 cutpoint in COPD guidelines remain to be seen [20]. Recent discussions illustrate that there currently is no consensus on this issue [21-23] and that there is a clear need for further evidence.

The aim of the study reported in this paper was to assess lung function decline in symptomatic middle-aged and elderly subjects identified as 'obstructive' according to either the fixed 0.70 FEV1/FVC cutpoint or an age- and gender-specific LLN cutpoint for this ratio. We also investigated whether our findings and conclusions would change when different sets of prediction equations are used to calculate LLN cutpoints for FEV1/FVC.

## Methods

### Study setting and cohort

This cohort study was based on all available spirometry tests from the period October 2001 to March 2010 from three regional primary care diagnostic centres in the Netherlands (the General Practice Laboratory Foundation Etten-Leur/Breda (SHL), the Diagnostic Centre Eindhoven (DCE), and the General Practice Laboratory East (SHO)). These diagnostic centres offer a range of diagnostic tests (including spirometry) and other healthcare services to hundreds of general practitioners (GPs) in the south-western and south-eastern parts of our country since the mid- or late nineteen-nineties. When a patient consults with respiratory symptoms and the GP suspects an underlying chronic respiratory condition (e.g., COPD or asthma), he or she can refer the patient to the diagnostic centre for spirometry testing. When a chronic respiratory condition is diagnosed or still suspected, the majority of patients enter the diagnostic centre's monitoring service and return for reassessment every six to twelve months.

Spirometry test results and accompanying demographic (gender, age), anthropometric (height, weight) and medical history information (self-reported smoking status and history, respiratory symptoms, medication) are recorded during each visit using a standardized electronic format. Every spirometry test is assessed by a respiratory consultant whose interpretation of the test and - if applicable - diagnostic advice is sent to the GP, together with the actual test results. Further details about the spirometry tests performed in the diagnostic centres are described elsewhere [16]. Since only routine lung function and respiratory medical history data were used for our analyses and the investigators had no access to the patients' medical records or information on patients' identity, no written informed consent was obtained.

### Subject selection and definitions for airflow obstruction

**Figure 1:**
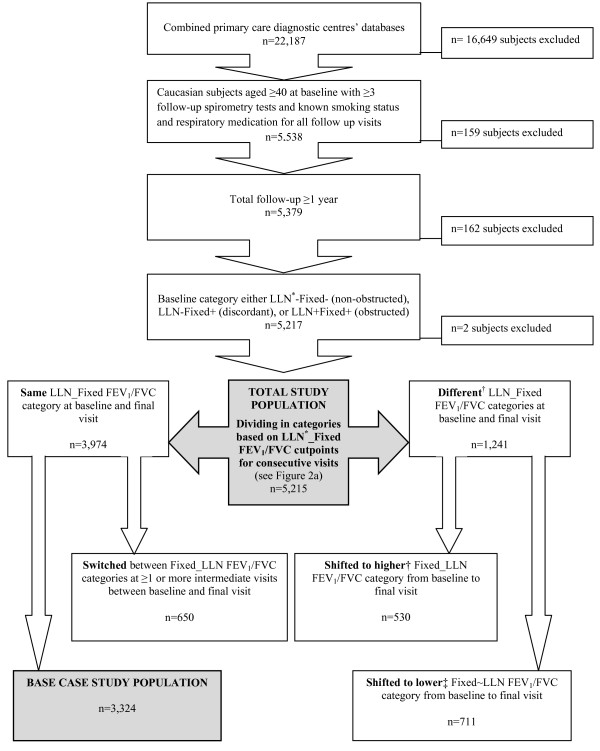
**Selection of study subjects from the initial primary care diagnostic centres' spirometry databases**. FEV1: forced expiratory volume in 1 s; FVC: forced vital capacity; LLN: lower limit of normal. * based on Swanney prediction equations for FEV1/FVC [6] † subgroups D, E, and F in Figure 2b ‡ subgroups A, B, and C in Figure 2b.

We selected all data from Caucasian subjects aged ≥ 40 years with complete data regarding height, history of cigarette smoking, and respiratory medication use for whom at least three postbronchodilator spirometry tests were available during a minimum follow-up of one year (see Figure 1). We used postbronchodilator FEV1/FVC values to determine whether or not airflow obstruction was present in the study subjects. The following two definitions for airflow obstruction applied:

1) Fixed cutpoint definition: postbronchodilator FEV1/FVC < 0.70. This is the definition for airflow obstruction that is currently recommended in clinical COPD guidelines [1,24].

2) LLN cutpoint definition: postbronchodilator FEV1/FVC below the subjects' age-specific LLN value [25]. When the resulting standard deviation (SD) score (also known as 'standardized Z score') was < -1.645, airflow obstruction was present according to this definition. This corresponds with the 5^th ^percentile.

The principal prediction equations used to calculate LLN values for FEV1/FVC were those recently published by Swanney et al, which have been derived from an appropriate Dutch general population cohort [6]. We also used several other LLN equations as alternatives to calculate age and gender specific cutpoints: European Community for Steel and Coal (ECSC) [25], Falaschetti *et al *[26], Brandli *et al *[27], Kuster *et al *[28], and Hankinson *et al *[29]. We selected these LLN equations from an extensive list of reference equations [6] based on the following criteria: Caucasian race; includes age > 40 years; published in the last ten years. One additional set of equations that was more recently published was added to the selection post-hoc [28].

### Categorization of airflow obstruction

Before further analysis we subdivided the study population into four categories based on the presence of airflow obstruction at baseline as defined by the LLN and the fixed 0.70 FEV1/FVC cutpoint definitions:

- 'LLN-Fixed-': absence of airflow obstruction according to both definitions (further referred to as '**non-obstructed**' subjects);

- 'LLN-Fixed+': absence of airflow obstruction according to the LLN definition, but presence of airflow obstruction according to the fixed definition (further referred to as '**discordant**' subjects);

- 'LLN + Fixed+': presence of airflow obstruction according to both definitions (further referred to as '**obstructed' **subjects);

- 'LLN + Fixed-': presence of airflow obstruction according to the LLN definition, but absence of airflow obstruction according to the fixed definition.

**Figure 2:**
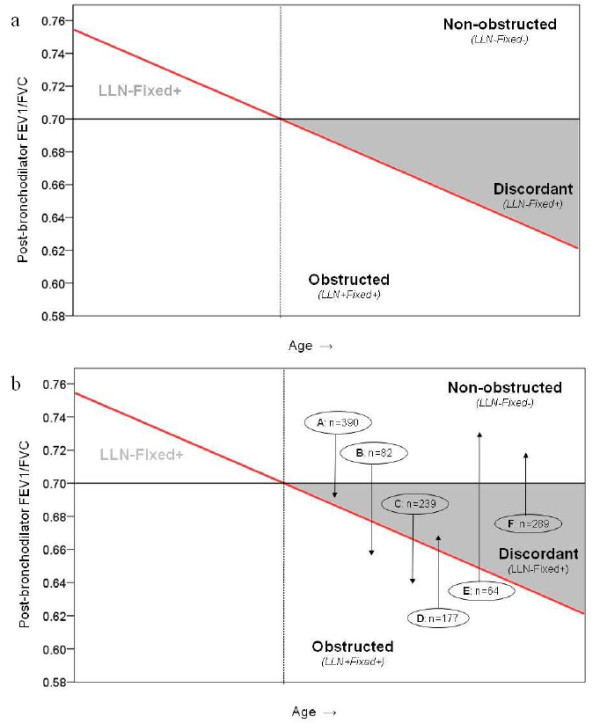
**A. Categories based on the lower limit of normal **[6]) **and fixed 0.70 FEV_1_/FVC definitions**. FEV1: forced expiratory volume in 1 s; FVC: forced vital capacity; LLN: lower limit of normal Red diagonal line: LLN FEV1/FVC cutpoint (position and slope of this line will vary depending on the reference equation used and the height and age of the subject); black horizontal line: fixed FEV1/FVC cutpoint at 0.70 LLN-Fixed-: absence of obstruction according to both LLN and Fixed FEV1/FVC cutpoints (**non-obstructed **subjects) LLN-Fixed+: absence of obstruction according to LLN cutpoint, but presence of obstruction according to fixed cutpoint (**discordant **subjects) LLN+Fixed+: presence of obstruction according to both LLN and Fixed FEV1/FVC cutpoints (**obstructed **subjects) LLN+Fixed-: presence of obstruction according to LLN cutpoint, but absence of obstruction according to fixed cutpoint. Only two subjects were present in this category, who were excluded from further analysis (see Figure 1) **B. Categories based on the lower limit of normal **[6]**and fixed 0.70 FEV_1_/FVC definitions, and subgroups of subjects who showed a downward shift (A, B and C, n = 711) or an upward shift (D, E and F, n = 530) between categories over time**. Subgroups A: no obstruction at baseline visit, obstruction according to fixed FEV1/FVC cutpoint at last available visit. B: no obstruction at baseline visit, obstruction according to both fixed and LLN FEV1/FVC cutpoints at last available visit. C: obstruction according to baseline FEV1/FVC cutpoint at first visit, obstruction according to both fixed and LLN cutpoints at last available visit. D: obstruction according to fixed and LLN FEV1/FVC cutpoints at baseline visit, obstruction according to fixed cutpoint at last available visit. E: obstruction according to fixed FEV1/FVC cutpoint at baseline visit, no obstruction at last available visit. F: obstruction according to fixed and LLN FEV1/FVC cutpoints at baseline visit, no obstruction at last available visit.

Figure 2a illustrates these categories. Only two subjects had obstruction according to the LLN definition but not according to the fixed 0.70 definition ('LLN + Fixed-'). These subjects were excluded from further analysis.

During the process of analysis we found that a substantial number of subjects (36% of the initial study cohort) shifted to another category after their initial visit. Because we considered consistency in classification to be essential for the 'proof of concept' that underlies the aim of this paper, we limited the analysis to subjects who were consistently classified in the same category (i.e., non-obstructed, discordant, or obstructed) throughout their entire follow-up ('base case population', see Figure 1).

### Outcomes and statistical analysis

The primary outcome to compare the clinical course of the subjects in the three categories was the annual rate of postbronchodilator FEV_1 _decline [30]. We analyzed prebronchodilator FEV_1 _decline and pre- and postbronchodilator FVC decline as secondary outcomes.

SAS^® ^Proprietary Software 9.2 (SAS Institute Inc., Cary, NC, USA) was used for all analyses. p < 0.05 was considered statistically significant. Baseline differences between the non-obstructed, discordant, and obstructed categories were tested with analysis of variance (Anova), Kruskal-Wallis, and Pearson Chi-square tests. A random coefficient regression model with random intercept and random slope was used to estimate the annual decline of postbronchodilator and prebronchodilator FEV1 and FVC in baseline smokers and non-smokers separately (PROC MIXED in SAS). Comparison of the discordant and obstructed categories was the principal part of the analyses, but we also compared lung function decline between discordant and non-obstructed subjects. We did not include age and gender in the regression models. Because of its known (but marginal) effect on FEV1 decline, use of inhaled corticosteroids (regardless of the dose being prescribed) during each subsequent visit was included in the model as a time-dependent dichotomous (yes/no inhaled corticosteroid use) covariate. As the proportion of subjects who reported to have changed smoking status during follow-up was small (4% of all baseline ex-smokers reported to have taken up smoking again, 5% of all baseline smokers reported to have stopped smoking) and very similar for the respective categories, we did not include changes in smoking status during follow-up in the respective regression models for smokers and non-smokers. In order to assess the sensitivity of our findings, we repeated the base case analysis after categorization of the study subjects using the selected alternative LLN prediction equations [6,26-29].

## Results

### Study subjects

The total study population consisted of 5,215 respiratory symptomatic subjects aged ≥ 40 years who had been referred for spirometry by their GP and for whom complete data for three measurements in at least one year were available (Figure 1). 1,241 subjects were in different categories based on the fixed and LLN definitions for airflow obstruction during their baseline and final visits. Tables 1 and 2 show baseline characteristics for these subjects. 650 subjects were in the same category during their baseline and final visits but had shifted between categories during intermediate visits. Ultimately, 3,324 subjects (64%) could be included in the base case analysis as they remained in the same category during all visits. Table 3 shows characteristics for the base case population and for the three defined categories.

**Table 1:** Baseline characteristics of subjects who showed a downward shift in FEV1/FVC category between their baseline and final visits

	Subgroup					
	
	A (n = 390)		B (n = 82)		C (n = 239)	
	
	LLN-Fixed- → LLN-Fixed+		LLN-Fixed- → LLN + Fixed+		LLN-Fixed + → LLN + Fixed+	
	**Non-smokers** **(n = 246)**	**Smokers** **(n = 144)**	**Non-smokers** **(n = 27)**	**Smokers** **(n = 55)**	**Non-smokers** **(n = 102)**	**Smokers** **(n = 137)**

Males, n (%)	133 (54)	65 (45)	14 (52)	16 (29)	80 (78)	79 (58)
Age, years (SD)	64.9 (11.0)	58.3 (9.3)	53.0 (10.3)	52.6 (9.1)	63.6 (9.2)	61.7 (9.0)
Baseline postbronchodilator FEV1/FVC (SD)	0.75 (0.04)	0.75 (0.04)	0.75 (0.05)	0.75 (0.05)	0.64 (0.04)	0.65 (0.04)
Baseline postbronchodilator FEV1, liters (SD)	2.33 (0.76)	2.33 (0.67)	2.28 (0.60)	2.10 (0.58)	2.22(0.68)	2.09 (0.66)
as % predicted* (SD)	78.0 (14.8)	76.8 (12.1)	72.8 (14.3)	71.6 (11.9)	68.8 (12.2)	67.6 (12.7)

See Figure 2b for further illustration of subgroups

FEV_1_: forced expiratory volume in 1 s; FVC: forced vital capacity; LNN: lower limit of normal; SD: standard deviation

* based on Swanney prediction equations [6]

**Table 2:** Baseline characteristics of subjects who showed an upward shift in FEV1/FVC category between their baseline and final visits

	Subgroup					
	
	D (n = 177)		E (n = 64)		F (n = 289)	
	**LLN + Fixed + → LLN-Fixed+**		**LLN + Fixed + → LLN-Fixed-**		**LLN-Fixed + → LLN-Fixed-**	

	**Non-smokers** **(n = 97)**	**Smokers** **(n = 80)**	**Non-smokers** **(n = 33)**	**Smokers** **(n = 31)**	**Non-smokers** **(n = 210)**	**Smokers** **(n = 79)**

Males, n (%)	65 (67)	44 (55)	16 (49)	8 (26)	105 (50)	42 (53)
Age, years (SD)	63.4 (10.3)	58.9 (10.0)	53.7 (11.2)	52.7 (7.7)	63.4 (9.7)	60 (10.6)
Baseline postbronchodilator FEV1/FVC (SD)	0.56 (0.06)	0.57 (0.06)	0.61 (0.06)	0.60 (0.06)	0.67(0.03)	0.67 (.02)
Baseline postbronchodilator FEV1, liters (SD)	1.86 (0.59)	1.99 (0.65)	2.08 (0.65)	1.87 (0.59)	2.26 (0.71)	2.24 (0.62)
as % predicted* (SD)	59.7 (13.2)	61.7 (14.0)	64.3 (15.4)	63.5 (14.0)	74.8. (12.2)	71.1 (11.3)

* based on Swanney prediction equations [6]

**Table 3:** Baseline characteristics for the base case study population (categories based on Swanney prediction equations [6])

	Total cohort (n = 3,324)	Non-obstructed (n = 2,406)	Discordant (n = 389)	Obstructed (n = 529)	p value
		LLN*-Fixed-	LLN*-Fixed+	LLN* + Fixed+	
Follow-up time, years (SD)	3.4 (1.4)	3.4 (1.4)	3.2 (1.4)	3.4 (1.5)	0.592
Males, n (%)	1,500 (45)	894 (37)	245 (63)	361 (68)	< 0.001
Age, mean (SD)	58.1 (11.0)	56.2 (10.5)	67.0 (10.1)	60.3 (10.2)	< 0.001
40-49, n (%)	918 (28)	798 (33)	30 (8)	90 (17)	
50-59, n (%)	1,003 (30)	758 (31)	66 (17)	179 (34)	
60-69, n (%)	827 (25)	553 (23)	118 (30)	156 (29)	
70-79, n (%)	512 (15)	277 (12)	144 (37)	91 (17)	
> 80, n (%)	64 (2)	20 (1)	31 (8)	13 (3)	< 0.001
Height, m	1.69 (0.1)	1.68 (0.1)	1.73 (0.1)	1.70 (0.1)	< 0.001
BMI, kg/m [2] (SD)	27.5 (5.0)	28.2 (5.2)	27.0 (4.2)	25.0 (4.0)	< 0.001
Smoking status, n (%)					
Never	956 (29)	864 (36)	59 (15)	33 (6)	
Former	1,360 (41)	964 (40)	192 (49)	204 (39)	
Current	1,008 (30)	578 (24)	138 (36)	292 (55)	< 0.001
Respiratory medication use, n (%)					
Short-acting bronchodilators	1,436 (43)	1,061 (44)	147 (38)	228 (43)	0.066
Long-acting bronchodilators	1,174 (35)	841 (35)	144 (37)	189 (36)	0.715
Inhaled corticosteroids	1,972 (59)	1,550 (64)	185 (48)	237 (45)	< 0.001
FEV1					
Prebronchodilator, liters (SD)	2.28 (0.74)	2.46 (0.71)	2.02 (0.65)	1.64 (0.51)	< 0.001
as % predicted* (SD)	74.6 (17.9)	81.1 (14.5)	65.3 (12.1)	51.7 (12.8)	< 0.001
Postbronchodilator, liters (SD)	2.46 (0.77)	2.63 (0.74)	2.21 (0.68)	1.85 (0.56)	< 0.001
as % predicted* (SD)	80.2 (17.4)	86.5 (14.1)	71.7 (12.3)	58.1 (13.2)	< 0.001
FVC					
Prebronchodilator, liters (SD)	3.23 (0.95)	3.22 (0.95)	3.24 (1.03)	3.26 (0.91)	0.708
Postbronchodilator, liters (SD)	3.35 (0.96)	3.30 (0.95)	3.43 (1.03)	3.53 (0.92)	< 0.001
FEV1/FVC					
Prebronchodilator (SD)	0.71 (0.12)	0.77 (0.07)	0.63 (0.05)	0.51 (0.08)	< 0.001
Postbronchodilator (SD)	0.74 (0.12)	0.80 (0.05)	0.65 (0.03)	0.52 (0.08)	< 0.001

BMI: body mass index; FEV1: forced expiratory volume in 1 s; FVC: forced vital capacity; LNN: lower limit of normal; SD: standard deviation

* based on Swanney prediction equations [6]

In the base case population the non-obstructed ('LLN-Fixed-') category comprised 2,406 subjects (72%), the obstructed ('LLN + Fixed + ') category 529 subjects (16%). 389 subjects (12% of all subjects and 42% of subjects with obstruction according to the fixed FEV1/FVC cutpoint) were classified as having airflow obstruction according to the fixed but not to the LLN FEV1/FVC cutpoint ('discordant' or LLN-Fixed + category). Mean number of spirometry tests per subject was 4.0 (SD 1.2) and mean follow-up was 3.4 (SD 1.4) years, with no differences between the categories (p = 0.592).

The non-obstructed category showed a higher proportion of females (63%, p < 0.001), never smokers (36%, p < 0.001), and inhaled corticosteroid users (64%, p < 0.001). With a mean age of 67.0 (SD 10.1) years the subjects in the discordant category were significantly older than those in the non-obstructed (56.2 (SD 10.5) years) and obstructed (60.3 (SD 10.2) years) categories (p < 0.001). With a mean postbronchodilator FEV1 percentage predicted of 71.7% the discordant subjects were in between the mean values observed in the other two categories (Table 3). The obstructed subjects were significantly more severe in terms of postbronchodilator FEV1 percentage predicted: 58.1% (p < 0.001).

In the non-obstructed category the respiratory consultants could not provide a clear diagnostic advice to the GP in 44% of subjects. Analysis of the non-obstructed subjects in whom a diagnostic advice was given showed that 76% of this category consisted of subjects with reversible obstruction or (probable) asthma, and ~2% of subjects with (probable) restriction. In the remaining 22% of non-obstructed subjects the consultants recommended additional diagnostic testing (e.g., bronchial provocation, full pulmonary function testing) and/or further follow-up in order to exclude underlying chronic respiratory morbidity.

### Rate of lung function decline

**Figure 3:**
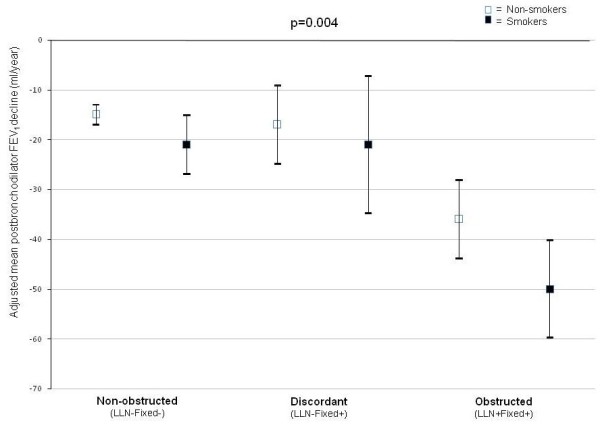
Differences* in postbronchodilator FEV1 decline for smokers and non-smokers in the non-obstructed, discordant, and obstructed categories^† ^for the base case population. Bars indicate 95% confidence intervals. FEV1: forced expiratory volume in 1 s; LLN: lower limit of normal. * adjusted for differences in inhaled corticosteroid use between the categories. † based on Swanney [6] prediction equations for FEV1/FVC.

Figure 3 shows the adjusted estimates of the mean annual postbronchodilator FEV1 decline (primary outcome) and 95% confidence intervals for base case population smokers and non-smokers in each category. In all three categories smokers showed a more rapid FEV1 decline compared to non-smokers. Rates of postbronchodilator FEV1 decline differed significantly between the three categories (p < 0.001). Table 4 shows that the smokers as well as the non-smokers in the discordant category showed a rate of postbronchodilator FEV1 decline which was very similar to that in the non-obstructed category (p = 0.973 for smokers, p = 0.624 for non-smokers), but only about half the rate observed in the obstructed category (p < 0.004 for smokers and p < 0.004 for non-smokers).

**Table 4:** Mean (standard error) results for the primary and the secondary outcomes in the three defined categories for current smokers and non-smokers in the base case population

		Category				
				
	Non-obstructed (n = 2,406)	Discordant (n = 389)	Obstructed (n = 529)	p-value*		
	**(LLN-Fixed-)**	**(LLN-Fixed+)**	**(LLN+Fixed+)**	**Overall difference** **between categories**	**LLN-Fixed+ *versus*** **LLN-Fixed-**	**LLN-Fixed+ *versus*** **LLN+Fixed+**

ΔFEV1 postbronchodilator, ml/year (SE)						
Smokers	-21 (3)	-21 (7)	-50 (5)	< 0.001	0.973	0.004
n	578	138	292			
Non-smokers	-15 (1)	-17 (4)	-36 (4)	< 0.001	0.624	0.004
n	1828	251	237			
ΔFEV1 prebronchodilator, ml/year (SE)						
Smokers	-13 (3)	-13 (7)	-50 (5)	< 0.001	0.998	< 0.001
n	578	138	292			
Non-smokers	-9 (2)	-14 (5)	-30 (5)	< 0.001	0.301	0.016
n	1828	251	237			
ΔFVC postbronchodilator, ml/year (SE)						
Smokers	-18 (5)	-17 (9)	-33 (6)	0.133	0.914	0.154
n	578	138	292			
Non-smokers	-15 (2)	-18 (6)	-22 (5)	0.420	0.555	0.647
n	1828	251	237			
ΔFVC prebronchodilator, ml/year (SE)						
Smokers	-12 (5)	-5 (10)	-46 (7)	< 0.001	0.544	0.001
n	578	138	292			
Non-smokers	-7 (2)	-15 (6)	-16 (6)	0.226	0.230	0.929
n	1828	251	237			

LNN: lower limit of normal; FEV1: forced expiratory volume in 1 s; FVC: forced vital capacity; Δ: change;

* between group differences were analyzed with a random coefficient regression model with random intercept and random slope and were adjusted for differences in inhaled corticosteroid use between the categories. Inhaled corticosteroid use was statistically significantly associated with lung function decline at p < 0.001 level in all regression models

Fixed: FEV_1_/FVC < 0.70; LLN based on Swanney prediction equations [6]

With regard to the secondary outcomes, analysis of prebronchodilator FEV1 values also indicated similarity in terms of decline between the discordant and non-obstructed categories (Table 4; p = 0.998 for smokers, p = 0.301 for non-smokers), but dissimilarity between the discordant and obstructed categories (p < 0.001 for smokers and p = 0.016 for non-smokers). Prebronchodilator FVC decline was statistically significantly different between the three categories in smokers only (p < 0.001), with a significant difference in decline between discordant (-5 ml/year) and obstructed (-46 ml/year) subjects (p < 0.001). Postbronchodilator FVC decline was not significantly differ between the respective categories.

### Sensitivity analysis with alternative lower limit of normal equations

Table 5 (for smokers) and 6 (for non-smokers) show the estimates of the mean annual postbronchodilator FEV1 decline when the non-obstructed, discordant and obstructed categories were based on alternative LLN equations. For all equations the overall differences in FEV1 decline between the categories were statistically significant (p < 0.001, Tables 5 and 6). For non-obstructed and obstructed smokers the mean values for the decline were rather similar for the different equations (ranging from -20 to -22 ml/year and -47 to -50 ml/year, respectively; Table 5). For smokers in the discordant category the estimates ranged from -4 ml/year (Brandli [27] equations) to -37 ml/year (Hankinson [29] equations). Postbronchodilator FEV1 decline in discordant and obstructed smokers differed significantly for the Brandli [27] equations (p < 0.001) and showed tendency towards statistical significance for the ECSC [25] (p = 0.094) and Kuster [28] (p = 0.087) equations (see Table 5).

**Table 5:** Sensitivity analysis for smokers: mean (SE) postbronchodilator FEV1 decline in ml/year in the three categories* based on alternative LLN prediction equations for the base case population

			Category*				
		
		Non-obstructed	Discordant	Obstructed	**p-value**^**†**^		
		**(LLN-Fixed-)**	**(LLN-Fixed+)**	**(LLN+Fixed+)**	**Overall difference** **between categories**	**LLN-Fixed+ *versus*** **LLN-Fixed-**	**LLN-Fixed+ *versus*** **LLN+Fixed+**

**Swanney **[6], **2008**	FEV1 decline	-21 (3)	-21 (7)	-50 (5)	< 0.001	0.973	< 0.001
	n	578	138	292			
ECSC [25], 1993	FEV1 decline	-22 (3)	-24 (15)	-50 (3)	< 0.001	0.870	0.094
	n	578	29	452			
Kuster [28], 2008	FEV1 decline	-20 (3)	-8 (20)	-48 (3)	< 0.001	0.599	0.087
	n	563	14	557			
Falaschetti [26], 2004	FEV1 decline	-21 (3)	-21 (18)	-47 (3)	< 0.001	0.985	0.153
	n	546	24	542			
Brandli [27], 2000	FEV1 decline	-21 (3)	-4 (11)	-49 (4)	< 0.001	0.026	< 0.001
	n	575	60	397			
Hankinson [29], 1999	FEV1 decline	-21 (3)	-37 (22)	-48 (3)	< 0.001	0.468	0.620
	n	569	18	523			

ECSC: European Community for Steel and Coal; FEV_1_: forced expiratory volume in 1 s; LLN: lower limit of normal; SD: standard deviation

* based on reference equations for LLN as indicated in first column

† between group differences were analyzed using random coefficient regression models with random intercept and random slope; adjustment for inhaled corticosteroid use

**Table 6:** Sensitivity analysis for non-smokers: mean (SE) postbronchodilator FEV1 decline in ml/year in the three categories* based on alternative LLN prediction equations for the base case population

		Category*					
		
		Non-obstructed	Discordant	Obstructed	**p-value**^**†**^		
		**(LLN-Fixed-)**	**(LLN-Fixed+)**	**(LLN+Fixed+)**	**Overall difference** **between categories**	**LLN-Fixed+ *versus*** **LLN-Fixed-**	**LLN-Fixed+ *versus*** **LLN+Fixed+**

**Swanney **[6], 2008	FEV1 decline	-15 (1)	-17 (4)	-36 (4)	< 0.001	0.624	< 0.001
	n	1,828	251	237			
ECSC [25], 1993	FEV1 decline	-15 (1)	-15 (8)	-28 (3)	< 0.001	0.975	0.132
	n	1,831	71	426			
Kuster [28], 2008	FEV1 decline	-15 (1)	-8 (12)	-26 (3)	< 0.001	0.076	0.009
	n	1,814	28	532			
Falaschetti [26], 2004	FEV1 decline	-15 (1)	-22 (8)	-27 (3)	< 0.001	0.376	0.521
	n	1,785	71	488			
Brandli [27], 2000	FEV1 decline	-15 (1)	-17 (6)	-31 (3)	< 0.001	0.746	0.036
	n	1,828	125	349			
Hankinson [29], 1999	FEV1 decline	-15 (1)	-7 (10)	-26 (3)	< 0.001	0.424	0.061
	n	1,816	46	482			

ECSC: European Community for Steel and Coal; FEV_1_: forced expiratory volume in 1 s; LLN: lower limit of normal; SD: standard deviation

* based on reference equations for LLN as indicated in first column

† between group differences were analyzed using random coefficient regression models with random intercept and random slope; adjustment for inhaled corticosteroid use

The estimates for FEV1 decline in non-obstructed non-smokers were identical for all alternative LLN equations (-15 ml/year; Table 6), and for obstructed non-smokers the estimates ranged from -26 to -31 ml/year. For non-smokers in the discordant category the estimates ranged from -7 ml/year (Hankinson [29] equations) to -22 ml/year (Falaschetti [26] equations). FEV1 decline in discordant and obstructed non-smokers differed significantly for the Kuster [28] (p = 0.009) and Brandli [27] (p = 0.036) equations, with tendency to statistical significance for the Hankinson [29] equations (p = 0.061, see Table 6).

## Discussion

In this 'proof of concept' study we were primarily interested in the rate of lung function decline in subjects identified as obstructive by the fixed 0.70 FEV1/FVC cutpoint but as non-obstructive by an age and gender specific LLN cutpoint for this ratio (the discordant or 'LLN-Fixed+' category). Analysis of our base case population showed that the annual postbronchodilator FEV1 decline in discordant subjects was very similar to subjects who were non-obstructive according to both definitions (i.e., LLN-Fixed- category), but also that it was less than half the rate of decline observed in subjects with FEV1/FVC values below their age-specific LLN cutpoint (obstructed or 'LLN + Fixed+' subjects). The difference in FEV1 decline between discordant and obstructed subjects was highly statistically significant. Use of alternative FEV1/FVC prediction equations generally showed that the annual postbronchodilator FEV1 decline in discordant subjects was lower compared with obstructed subjects. However, the use of different prediction equations lead to variable numbers of subjects in the respective categories, resulting in different estimates of FEV1 decline, variances and levels of statistical significance. It is therefore important that the most appropriate prediction equations for the study population are used to provide unbiased estimates of FEV1 decline and correct conclusions.

It is important to realize that the non-obstructed (LLN-Fixed-) category did not consist of 'healthy' subjects in respiratory terms, even while the respiratory consultants could not provide clear diagnostic advice in 44% of non-obstructed subjects. As the proportion of current and former smokers was rather high in these subjects (~90%), it is likely that the GPs included them in the respiratory monitoring service because of their increased risk for developing a chronic respiratory condition or persistence of respiratory symptoms (in the absence of concomitant spirometric abnormalities).

### Comparison with existing literature

Several recent studies have looked at discrepancies between fixed and LLN definitions for airflow obstruction and reported high rates of 'false-positive' diagnostic interpretations when the 0.70 fixed cutpoint is used [10-12,14,31]. False-positive interpretations may cause erroneous diagnoses in individuals and inflate COPD population prevalence rates [32]. Until now only few studies have looked at the course of clinical markers of COPD prognosis or outcome in relation to criteria for defining airflow obstruction. In a previous study from our group we have shown that in a primary care cohort of undiagnosed adults lung function below the normal range and early respiratory signs predicted the development and progression of COPD in the next five years [33]. Garcio-Rio and colleagues recently reported that subjects aged 40-80 who were 'over-diagnosed' with COPD by the fixed 0.70 ratio experienced worse quality of life compared to non-COPD subjects, but had similar exercise capacity and frequency of exacerbations [34]. Mannino and colleagues reported that symptomatic subjects aged ≥ 65 years who were classified as non-obstructive with a FEV1/FVC cutpoint based on LLN but as obstructive when the 0.70 cutpoint was applied, showed an increased risk of hospitalizations and death during a decade or more of follow-up [17,19]. However, these subjects were compared with healthy individuals without respiratory symptoms, and therefore the findings from this study do not actually address the issue of defining airflow obstruction for diagnostic purposes in subjects who seek medical attention for respiratory symptoms. Another important difference between the Mannino [17] study and our study is the age of the study population: while Mannino included only elderly subjects, we followed the lower age limit of 40 years for considering a diagnosis of COPD [1], and therefore studied a population in which a substantial number of subjects was younger than 65 years. Earlier epidemiologic research already showed that (in males) low FEV1/FVC is associated with a higher risk of all-cause mortality, after controlling for age, smoking, BMI, education, and respiratory diseases [35].

The use of a fixed cutpoint also causes misclassification of airflow obstruction in younger adults (i.e., aged < 45 years), as has recently been shown by Cerveri and colleagues [18]. In this age group the fixed 0.70 FEV1/FVC cutpoint identified less than 50% of subjects with evidence of airflow obstruction according to LLN cutpoints. These misclassified (or 'underdiagnosed') subjects were more likely to develop COPD in the following nine years, and had higher respiratory related healthcare than subjects without airflow obstruction according to the LLN cutpoints. Although we did not include subjects under the age of 40 in our study, our findings logically follow from those reported by Cerveri [18]: subjects who already have an FEV1/FVC value below their LLN cutpoint at younger age (which may still be above the fixed 0.70 FEV1/FVC cutpoint) must have an accelerated lung function decline in order to develop COPD in their next decades of life. Our observations indicate that the accelerated decline in subjects with below-LLN FEV1/FVC values seem to continue after the age of 40.

In their recent review, Soriano and colleagues acknowledged the fact that overestimation of airflow obstruction with the fixed FEV1/FVC ratio becomes more problematic with increasing age, but also state that the incremental benefits of changing the recommended fixed 0.70 cutpoint in the COPD guidelines remain to be seen [20]. Our study shows that the benefit of using age-specific FEV1/FVC cutpoints (based on lower limits of normal) is the provision of better discrimination between subjects who do show an accelerated lung function decline - the hallmark of prognosis in patients with COPD - and those who do not.

### Strengths and limitations

One of the particular strengths of our study is the large number of respiratory symptomatic subjects (3,324 in the base case population) with repeated postbronchodilator spirometry tests that could be included in our analysis. This enabled us to subdivide our study cohort into three categories based on the fixed and LLN definitions for airflow obstruction, while each category still contained a substantial number of subjects. Because we used routine data from primary care diagnostic centres, the subjects included in our analysis are a truly representative sample of the primary care patient population with a condition that has the GP refer them. It is important to realize that the ongoing debate about the most appropriate way to define airflow obstruction [21,22] is especially relevant for primary care physicians, as they are often confronted with middle-aged and elderly subjects who present with respiratory symptoms and who may or may not have COPD, asthma or another (respiratory) condition. The outcome of a spirometry test - often the only diagnostic procedure available to assess lung function in primary care - will direct their decision-making with regard to diagnosis and treatment, and use of an inappropriate cutpoint will misinform their decisions.

Because the goal of our study was to compare three clearly defined and consistent groups of subjects based on fixed and LLN definitions for airflow obstruction, we had to exclude those who shifted between categories during follow-up (about one third of the total study population) from the base case analysis. The finding that one off spirometry does not seem to be sufficient to rule in or out airflow obstruction in a substantial proportion of primary care patients is an important one, and suggests that GPs should not base a (COPD) diagnosis on a single spirometry test. On the other hand, excluding these subjects clearly comes at the cost of less generalizability. Therefore, our base case analysis should be seen as a 'proof of concept' study regarding the presumption that when diagnosing COPD, it is more appropriate to use age and gender specific cutpoints for the FEV1/FVC than it is to use the 'one size fits all' fixed 0.70 cutpoint. A final limitation of our study is the fact that we could not formally verify the diagnostic labels that underlie the treatment that had been initiated by the GP. Although we have looked at the interpretations from and diagnostic advice given by the respiratory consultants (which has shown to be a valid approach [34]), we did not have access to the GPs' medical records of the study subjects.

It is inevitable that if one takes certain individual characteristics into account when classifying subjects in categories (in this case gender and age, as these two factors determine the LLN cutpoint for each subject) the respective categories will differ on these particular characteristics (see Table 3). Thus, adjusting our regression models for the differences in age and gender between the categories would not be appropriate in this case: these characteristics cannot be considered as 'confounders' because they have deliberately been used to categorize subjects.

## Conclusions

We conclude that the use of the fixed 0.70 cutpoint for the FEV1/FVC ratio to define airflow obstruction does not seem to be an appropriate choice for primary care. Middle-aged and elderly respiratory symptomatic smokers as well as non-smokers who have values below this fixed FEV1/FVC cutpoint but above their age and gender specific lower limit of normal value, show about half the rate of lung function decline as that observed in those who are below their lower limit of normal value for the ratio. The rate of decline in these 'overdiagnosed' subjects does not differ from the decline observed in subjects without airflow obstruction, although the result is dependent on which FEV1/FVC prediction equation is used. Perseverance in recommending the use of the fixed cutpoint in clinical COPD guidelines seems to lead to a high rate of false-positive interpretations of obstructive airways disease in middle-aged and elderly subjects in primary care, and will inflate prevalence figures for COPD in population studies.

## Abbreviations

Anova: analysis of variance; COPD: chronic obstructive pulmonary disease; DCE: Diagnostics for U; ECSC: European Community for Steel and Coal; FEV1: forced expiratory volume in 1 second; FVC: forced vital capacity; GP: general practitioner; LLN: lower limit of normal; SD: standard deviation; SE: standard error; SHL: General Practice Laboratory Foundation Etten-Leur/Breda; SHO: General Practice Laboratory East.

## Competing interests

At the time of the study IJS acted as a paid consultant for the SHL primary care diagnostic centre, which has provided anonymous data for this study. BPT acts as a paid consultant for the SHO primary care diagnostic centre, which has provided anonymous data for this study. AEL is medical director of the DCE primary care diagnostic centre, which has provided anonymous data for this study. TRS received a research grant of Euro 45,000 from Boehringer Ingelheim for extracting, merging and maintaining datasets from the primary care diagnostic centres. The other authors have declared that they have no competing interests.

## Authors' contributions

The contributions of the authors have been as follows. RPA has been involved in the conception and design of the study, has conducted the statistical analyses, has contributed to the interpretation of the data, and has critically revised the article for important intellectual content. MAB has been involved in the conception and design of the study, selected data of eligible study subject, and prepared the draft version of the article. IJS, AEL, BPT, YFH and C van W have been involved in the conception and design of the study, have contributed to the interpretation of the data, and have critically revised the article for important intellectual content. TRS initiated and designed the study, has contributed to the interpretation of the data, has critically revised the article for important intellectual content, and had the overall supervision of the study. Dr. Schermer is the guarantor of the paper, taking responsibility for the integrity of the work as a whole, from inception to published article. All authors read and approved the final manuscript.

## Pre-publication history

The pre-publication history for this paper can be accessed here:

http://www.biomedcentral.com/1471-2466/12/12/prepub
